# The mitogenome diversity of Alpine Rendena cattle: new clues on its maternal origin and the complex substructure of haplogroup T3

**DOI:** 10.1186/s12711-026-01063-8

**Published:** 2026-07-01

**Authors:** Irene Cardinali, Anna Tommasi, Giacomo Villani, Nicola Rambaldi Migliore, Erika Partel, Elisa Somenzi, Simone Morabito, Ana Maria Chero Osorio, Rosalinda Di Gerlando, Anna Olivieri, Licia Colli, Heidi Christine Hauffe, Paolo Ajmone Marsan, Alessandro Achilli, Antonio Torroni, Hovirag Lancioni

**Affiliations:** 1 Department of Chemistry, Biology and Biotechnology, University of Perugia, Via Elce Di Sotto, 06123 Perugia, Italy; 2 Department of Biology and Biotechnology “Lazzaro Spallanzani”, University of Pavia, Via Ferrata 9, 27100 Pavia, Italy; 3 Fondazione E. Mach, Centro Trasferimento Tecnologico, Unità Risorse Foraggere e Produzioni Zootecniche, San Michele all’Adige, Via E. Mach 1, 38098 San Michele all’Adige, TN Italy; 4 Department of Animal Science, Food and Nutrition, Università Cattolica del Sacro Cuore, Via Emilia Parmense 84, 29122 Piacenza, Italy; 5 IRCCS Mondino Foundation, 27100 Pavia, Italy; 6 National Biodiversity Future Center (NBFC), Piazza Marina 61, 90133 Palermo, Italy; 7 BioDNA Research Centre on Biodiversity and Ancient DNA, Università Cattolica del Sacro Cuore, Via Emilia Parmense 84, 29122 Piacenza, Italy; 8 Fondazione E. Mach, Research and Innovation Centre, Conservation Genomics Research Unit, San Michele all’Adige, Via E. Mach 1, 38098 San Michele all’Adige, TN Italy

## Abstract

**Background:**

Val Rendena, an isolated Alpine valley in northern Italy, is home to an autochthonous, dual-purpose cattle breed with unique historical and morphological traits, which has been preserved by local breeders despite severe epidemics since the 1700s. While previous genome-wide studies identified signatures of selection in Rendena cattle, little is known about its evolutionary history. To address this issue, we analyzed complete mitogenomes from 137 Rendena individuals, selected to represent the majority of maternal lineages across the breed, as well as mitogenomes from 31 Alpine Grey individuals, purportedly closely related to Rendena cattle.

**Results:**

We identified 86 distinct mitochondrial DNA (mtDNA) haplotypes in the Rendena breed, indicating a high haplotype diversity (Hd = 0.986). Phylogenetic analyses revealed that virtually all samples belong to the T macro-haplogroup (T3 = 91%; T2 = 7%; T5 = 1%), with only one falling within the Q1 lineage. The comparison with the 31 Alpine Grey mitogenomes (27 haplotypes; Hd = 0.989) revealed a strong genetic proximity of the two Alpine cattle populations, suggesting either a recent common ancestry or historical maternal gene flow. The presence of rare haplogroups (T5 and Q1) combined with a high overall mtDNA diversity suggests a complex history of the Rendena breed. Notably, the analysis of Rendena mtDNA variation within a West-Eurasian context revealed an ancestral link with the Balkans. However, no haplotype sharing was observed. This supports the uniqueness of the Rendena maternal gene pool. Finally, the high frequency of haplogroup T3 mitogenomes in our survey allowed us to re-assess and refine the global phylogeny of this haplogroup, revealing evidence of population structuring within the Rendena breed.

**Conclusions:**

Complete mitogenomes reveal that both Rendena and Alpine Grey cattle harbor high maternal diversity and preserve rare, ancient taurine lineages. The refined phylogeny of T3 demonstrates that this dominant European haplogroup is far more structured than previously recognized, reflecting complex post-domestication dispersal and regional differentiation. These findings underscore the value of local, endangered breeds as reservoirs of unique genetic variation and highlight the importance of their conservation for understanding cattle evolutionary history.

**Supplementary Information:**

The online version contains supplementary material available at 10.1186/s12711-026-01063-8.

## Background

**Fig. 1:**
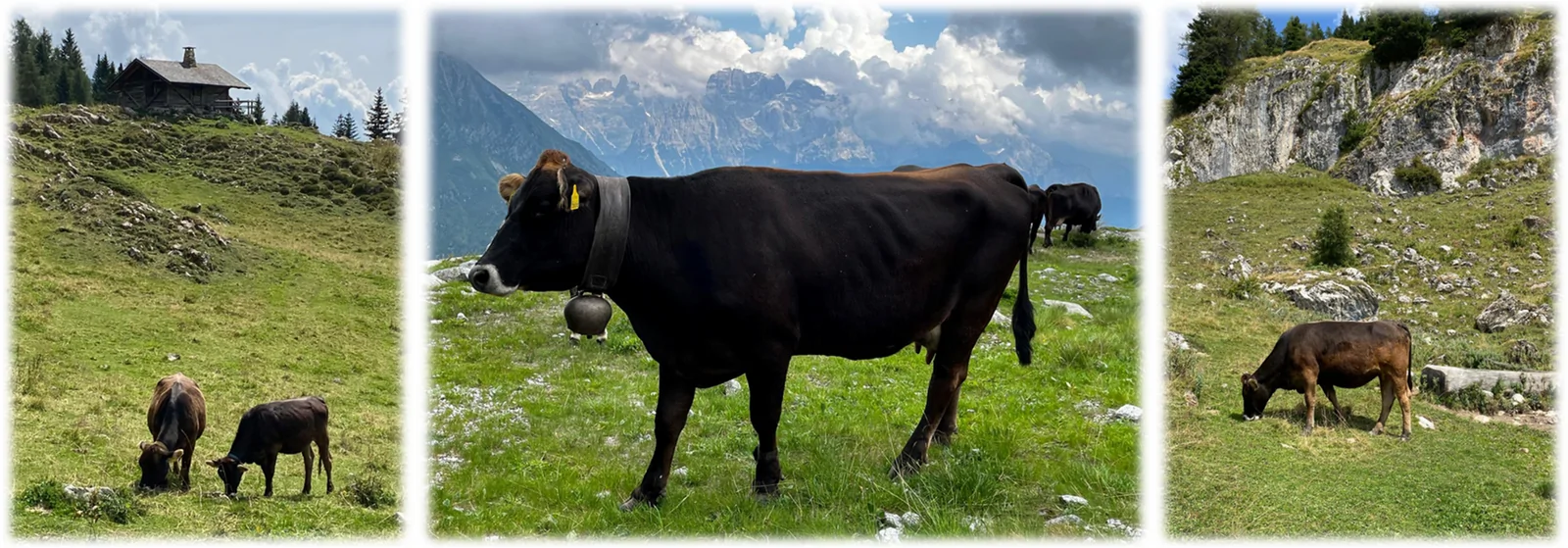
Typical Rendena cows in their native Valley (northern Italy)

As one of the highest and most extensive mountain ranges in Europe, the Alps represent a rich reservoir of agricultural biodiversity and ancient pastoral traditions. The region is characterized by a wide variety of cattle breeds, each with peculiar characteristics related to local agricultural practices and adaptation to the harsh mountain environment. Most of these breeds are dual-purpose and autochthonous, with limited distributions in marginal areas, including Rendena cattle [1], a breed characterized by a dark brown to nearly black coat (Fig. 1).

Distinctive breed features include ivory-colored hairs inside the ears, a lighter dorsal stripe along the loins, and light horns that are white at the base with black tips. The muzzle is slate-colored with a typical pale fringe. With only about 6000 animals (A.N.A.R.E.; www.anare.it*)*, this breed is considered ‘vulnerable to extinction’ (www.fao.org/dad-is) and native to the homonymous Italian Alpine valley located between the Adamello and Brenta mountain groups in the Province of Trento (northeastern Italy). The breed was first documented at the beginning of the 1700s [2], when a plague struck humans and their cattle in Val Rendena, forcing farmers to restock with imported Swiss bovines. Before the end of the eighteenth century, the restocking program was interrupted, and the Rendena became the most common dairy breed in the Valley by the end of the nineteenth century [2]. Since then, the breed spread to various valleys of Trentino and throughout Veneto. In the two decades between the two World Wars, the Italian government decreed local breeds to be replaced with more productive ones, such as the Original Braunvieh. However, although the population size of the Rendena decreased substantially, it survived mainly in the Rendena Valley, thanks to local farmers who continued to raise their native bovines without interbreeding, in the face of the authoritarian measures. Strategies for breed improvement in cattle usually focus on the selection of bulls to be crossbred with autochthonous cows [3], and a recent study on SNP data from the nuclear genome of Rendena cattle highlighted some unique genetic features [4]. We therefore conducted an analysis of maternally-transmitted mtDNA variation in Rendena cattle living in their ancestral valley to better characterize the genetic background of this breed.

The current mtDNA diversity in modern cattle breeds has been influenced by interbreeding [5, 6], and most available mtDNA data concerning native West-Eurasian cattle breeds derive from phylogenetic studies focused on the mtDNA control region [7–17], although there are also studies employing entire mitogenomes [18–28] or based on the identification of mtDNA polymorphisms from genome-wide analyses [6]. To date, the geographical distribution of West-Eurasian cattle breed maternal ancestry reflects past human migration pathways, with Europe populated by taurine cattle from the Middle East and some southern European breeds reflecting influences from both African taurine and indicine lineages [29–32].

Analyses of modern cattle mitogenomes have revealed several founder lineages: T, one of the major haplogroups; P, a possible marker of post-glacial expansion from Europe to Asia [33, 34]; R, which could represent a secondary localized domestication event of *Bos primigenius* or post-domestication hybridization with local aurochs either in Italy or North Africa [21, 35]; Q, belonging to the initial near eastern domesticated maternal pool; I, derived from *Bos indicus* [18, 36]; and a significant internal variation within haplogroup T (T1, T2, T3, T4, T5, and T6). Among the T sub-lineages, T3 is the most widespread in modern European cattle, since it predominantly accompanied the Neolithic expansion from the Fertile Crescent through Anatolia and the Balkans into continental Europe [13, 21]. The second most widespread lineage is T1, frequent in Africa, and present in the Iberian and Italian Peninsulas [22], as well as the Near East, followed by T2 and T5 [27]. Other highly divergent haplogroups, namely E, G, C, and K, have only been identified in ancient auroch specimens from Europe (E and G, from the Early Neolithic and the Pleistocene, respectively) and Central Asia (C and K, from the Holocene) [37, 38].

Even though the above results suggest a rich cattle biodiversity, genetic drift and human-mediated selection have progressively decreased the genetic variability and the effective population size in several breeds [39, 40], above all threatening local breeds which represent a valuable reservoir of genetic diversity [41]. On these premises, the aim of our study was to (i) shed light on the matrilineal origin of Rendena cattle and verify whether its genomic peculiarities were also present in the mitogenome; (ii) evaluate the genetic affinity previously observed by Senczuk and colleagues [42] between Rendena and another endangered Alpine cattle breed (Alpine Grey), and compare them with previously published mitogenomes of other West-Eurasian breeds; (iii) refine the global phylogeny of mtDNA haplogroup T3, the most common in Europe.

## Methods

Blood samples were collected by a licensed veterinarian during annual screening campaigns following Italian and European legislation on animal welfare (D.Lgs n. 146/2001, Council Directive 98/58/CE), and according to the European directive 2010/63. All experimental protocols were approved by the Ethics Committee for Clinical Experimentation of the University of Perugia (protocol no. 51/2023, October 10, 2023).

Whole blood was sampled from 140 Rendena (REN) dairy cows selected from official breeding records to avoid related animals and represent the main maternal lines of the breed (140 in total). DNA was extracted as previously reported [4]. A set of 32 Alpine Grey (GAL) individuals, randomly sampled from four different farms (Primiero, Fiemme, Rabbi and Sole Valleys), was added to the REN dataset, to evaluate (from a female perspective) the genetic affinity between the two breeds, and to address the gap of complete mtDNA data for Alpine Grey cattle. The entire mtDNA of each individual was amplified with Long-Range PCR as previously published in [24], and the mtDNA sequences were obtained using Illumina MiSeq technology.

Raw sequence processing included quality control with FastQC [43]; FASTQ files were then trimmed with Trim_galore v0.6.4_dev [44] to remove the adapters and the bases at the end of each read with a Phred quality score lower than 30. Short reads were aligned to the bovine mitochondrial sequence V00654 [45], here named BRS, which differs from the NC006853 in the following nucleotide positions (nps): 2536, 3559, 3560, 4497, 4537, 5718, 5754, 5853, 7994, 9682, 11,233, 12,158, 13,310, 15,510, 16,042, 16,093, using BWA [46] with the specific mem algorithm. Then the BAM files were filtered to retain only properly mapped reads with mapping quality ≥ 30 and were subsequently sorted using SAMtools [47]. Duplicates were removed using Picard’s MarkDuplicates [48] and BAM files were downscaled according to their average depth with the VariantBam tool [49]. The variants were called using Mutect2 implemented in the Genome Analysis Tool Kit (GATK) [50]. Variants with a frequency lower than 15% in our samples were removed with FilterMutectCalls (GATK package) and further filtered using BCFtools [47] to split multiallelic sites. Consensus sequence was then obtained using BCFtools. The IGV (Integrative Genomics Viewer) software [51] was used to visualize the BAM files (aligned to the V00654 and produced by the sequencing machine aligning software) and to verify specific mutational differences throughout the entire mitochondrial genome as well as heteroplasmies (mutations with a frequency between 15% and 85%). Although lower-frequency variants may represent true heteroplasmy, a conservative threshold was applied to ensure the reliability of variant calling. This approach is consistent with previous studies on mtDNA variation that apply similar or slightly lower thresholds to ensure robustness in heteroplasmy detection [52, 53, as examples]. The preliminary assessment of coding variation was performed using Geneious Prime v.2026.0.02 (www.geneious.com), which allowed us to verify the consistency of mutational motifs across mitogenomes and to contextualize variants within mtDNA coding regions. The quality of mitogenome sequences was manually checked, through the visualization of the original BAM files in IGV software (v.2.3.72) [51], and three REN and one GAL dirty sequences were excluded from the analyses. To exclude hypervariable C-stretches, we considered for the control region only nps 1 to 215, 222 to 351 and 363 to 16,338; the final haplotypes were classified into haplogroups according to the accepted cattle phylogeny (Additional file 1: Table S1).

Phylogenetic relationships between the 137 Rendena and 31 Alpine Grey mitogenomes were evaluated using BEAST v2.7.8 (Bayesian Evolutionary Analysis of Sampling Trees) [54], by employing the Hasegawa-Kishino-Yano (HKY) substitution model with gamma-distributed rate variation among sites and a relaxed molecular clock [24, 55]. The tree was rooted with *Bos grunniens* (NC_006380) and the mutational distances were converted into years using the substitution rate of one mutation every 3,172 years [18]. The Bayesian skyline plots (BSPs) [56] were obtained using a P mitogenome (DQ124389) as an outgroup. One hundred million iterations were run, with samples drawn every 10,000 Markov chain Monte Carlo (MCMC) steps, after a discarded burn-in of 10,000,000 steps. Major subclades in our samples were considered monophyletic in the analysis. The BSPs were visualized with Tracer v1.7.2.

Our Rendena and Alpine Grey mitogenomes were then compared with 635 mitogenomes of *Bos taurus* samples from across Western Eurasia retrieved in GenBank, as well as 30 mitogenomes of Original Braunvieh downloaded from Run 8 of the 1000 Bull Genomes Project, to test the hypothesis suggested by Somenzi and colleagues about the origin of the Rendena breed [4] (Additional file 1: Table S2).

To detail the complex substructure of haplogroup T3, a most parsimonious (MP) tree was generated with an updated version of mtPhyl v.5.003 [57] by including all published West-Eurasian mitogenomes (*N* = 480) and our 154 ones (125 REN and 29 GAL) belonging to haplogroup T3 (Additional file 1: Table S2). The Maximum Parsimony (MP) tree was checked with MEGA v.12.0.10 software to visualize the evolutionary relationships among West-Eurasian T3 mitogenomes.

## Results

### MtDNA haplotype analysis

The mtDNA variability of Rendena cattle was evaluated through the analysis of the entire mitogenome (16638 bp) of 137 individuals sampled as previously described [4]. The sequence alignment revealed 326 polymorphic sites (S), defining a total of 86 haplotypes. Considering a threshold of 15%, we found 12 heteroplasmic sites (nps 2685, 6201, 6637, 8365, 9711, 10292, 11287, 13415, 13908, 15273, 15417 and 16057). Nucleotide diversity (π) was estimated as 0.00074, while haplotype diversity (Hd) was 0.986, much higher than those found in other local breeds [6, 15, 58, 59], probably because our sampling strategy increased the representation of Rendena maternal lines.

Among the 137 mitogenomes, 62 presented unique haplotypes, while the remaining 24 haplotypes were shared between two or more samples (Additional file 1: Table S1).

A set of 31 Alpine Grey (GAL) samples was added to the REN dataset to evaluate the genetic links between the two breeds. The survey of their mitogenomes revealed 27 different haplotypes, with 132 polymorphic sites, Hd = 0.989 and π = 0.00071.

### MtDNA haplogroup classification and comparison with other West-Eurasian breeds

**Fig. 2:**
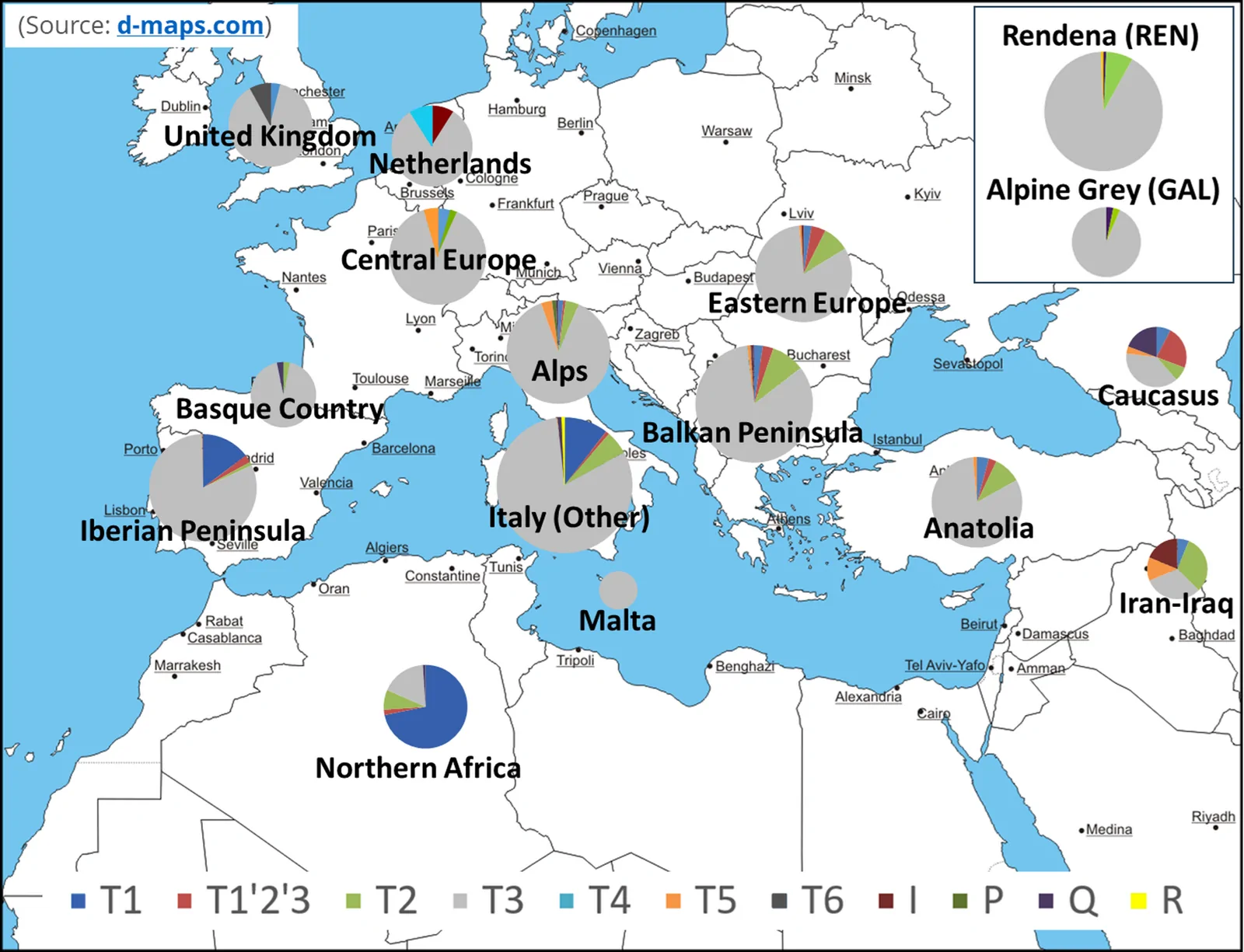
Frequency distributions of cattle mtDNA haplogroups in Western Eurasia and the Mediterranean area. See Additional File 1 Table S2 for frequency details

Haplogroup classification highlighted that all our REN samples but one belong to the primarily domesticated T macro-haplogroup, by far the most common in almost all modern taurine breeds. Within T, 128 REN mitogenomes (91%) were T3, the dominant maternal lineage in Europe [6, 18, 27, 60], ten (7%) belonged to haplogroup T2 and one (1%) to T5. Only one sample (1%) belonged to a non-T haplogroup (REN140), classified as Q1. Also, almost all of the 31 GAL mitogenomes were T3 (*N* = 29; 94%). The two remaining samples were T2 and Q1. Therefore, neither P nor R mitogenomes were found (Fig. 2, Additional file 1: Table S2).

To frame the mtDNA variation of the Rendena breed within a larger context, our sequences were compared with 635 mitogenomes of *B. taurus* samples from Western Eurasia and 30 Original Braunvieh mitogenomes. The geographic distribution of all mtDNA haplogroups in Western Eurasia and the Mediterranean area is shown in Fig. 2, highlighting the general prevalence of haplogroup T3, except in North Africa.

### Phylogenetic analysis

Four partial trees, one for each haplogroup (Q1, T2, T3, and T5) identified in our REN and GAL samples, were built by including all published mitogenomes belonging to these haplogroups from all breeds across different geographic areas (Additional file 2: Figs. S1–S4). The Q tree showed that one published mitogenome (from the Chianina breed) is close to our sample REN140, differing by one mutation (np 12480). In addition, our GAL005 is identical to the other two published Alpine Grey mitogenomes (Additional file 2: Fig. S1).

Among our samples, two with T2 haplotypes were found. The partial T2 tree highlighted a maternal proximity of one of these haplotypes found in REN063 and EU177661 (from [18]) to Iranian cattle, and the other (found in nine REN and one GAL samples; Additional file 1: Table S1) to the Dukajini Busha (Additional file 2: Fig. S2), a breed native to the Balkan Peninsula, thus further suggesting genetic inputs from this region into the Alps. The Rendena sample belonging to haplogroup T5 (REN115) had a unique haplotype, which in the MP tree is quite close to those found in other breeds from northern Italy [18] (Additional file 2: Fig. S3).

Most of the REN (*N* = 128) and GAL (*N* = 29) individuals investigated here belonged to the predominant T3 haplogroup, widespread among European cattle breeds (Fig. 2), even though among all our T3 samples (*N* = 157) only REN186 shares a mtDNA haplotype with another published T3 mitogenome (MZ901423; Tux-Zillertaler cattle breed) (Additional file 2: Fig. S4). The comparison with published T3 mtDNAs highlights a genetic proximity between Rendena and breeds from the Alps, Balkans and Iberian and Italian Peninsulas (Additional file 2: Fig. S4).

**Fig. 3:**
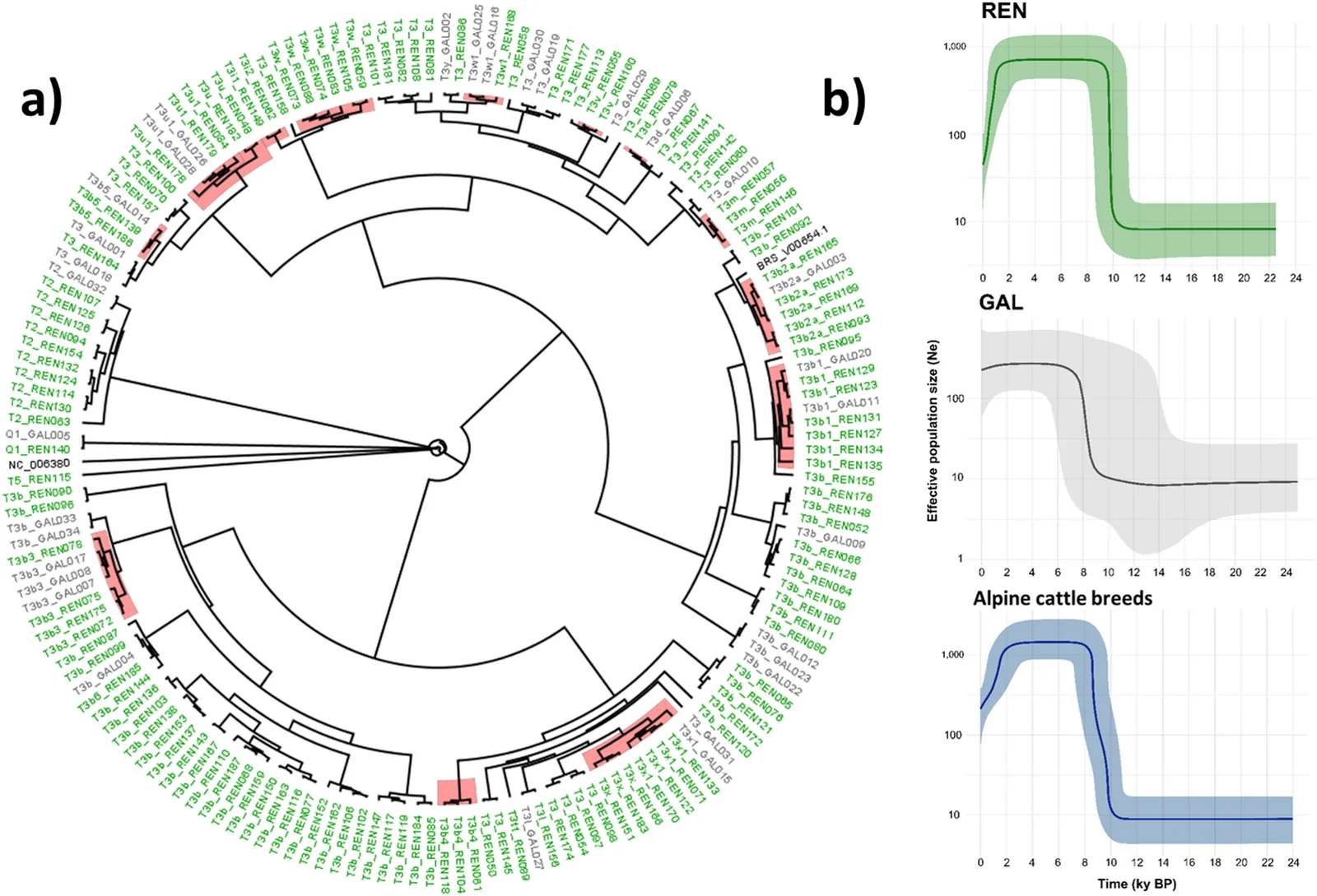
Phylogenetic tree and demographic trends obtained from Rendena (REN) and Alpine Grey (GAL) mitogenome data. **a** Maximum Parsimony (MP) tree encompassing 137 REN (green) and 31 GAL (grey) mitogenomes. The tree was rooted with a *Bos grunniens* mitogenome (NC_006380). Newly identified sub-clades of haplogroup T3 are shaded in light orange. **b** Bayesian skyline plots (BSPs) displaying changes in the effective population size (Ne) over time for REN, GAL and all the Alpine cattle breeds analysed in this study (including published mitogenomes; Additional file 1: Table S2). The x-axis represents time before present (kilo years), and the y-axis represents Ne on a log scale. Solid lines indicate median estimates, and shaded areas represent the 95% highest posterior density (HPD) intervals

The phylogenetic relationships of the two Alpine breeds analyzed here are shown in a MP tree, revealing a separation between T3 and non-T3 haplogroups (Fig. 3a).

To further assess the trend of population expansions that might have involved the Rendena Valley, Bayesian Skyline Plots (BSPs) were obtained on the single breeds and on all the Alpine cattle breeds including published mitogenomes (*N* = 241; Fig. 3b, Additional file 1: Table S2). For the REN breed, a rapid and pronounced expansion can be observed around 10 ky BP, followed by a plateau period of elevated Ne, suggesting a sustained large population size after the initial expansion. Then, around one thousand years ago, REN cattle suffered a marked decline, reflecting a bottleneck or reduction in population size. The same expansion around the Neolithic (10 ky BP) can also be observed in the BSP of GAL mitogenomes, whereas the later reduction of the effective population size in REN is absent in GAL (Fig. 3b). Nevertheless, when considering all the Alpine cattle breeds including published mitogenomes (*N* = 241), the BSP shows a trend similar to that of REN, with a Ne decline around 2.5 ky BP (Fig. 3b, Additional file 1: Table S2).

### Refinement of the substructure of haplogroup T3

**Fig. 4:**
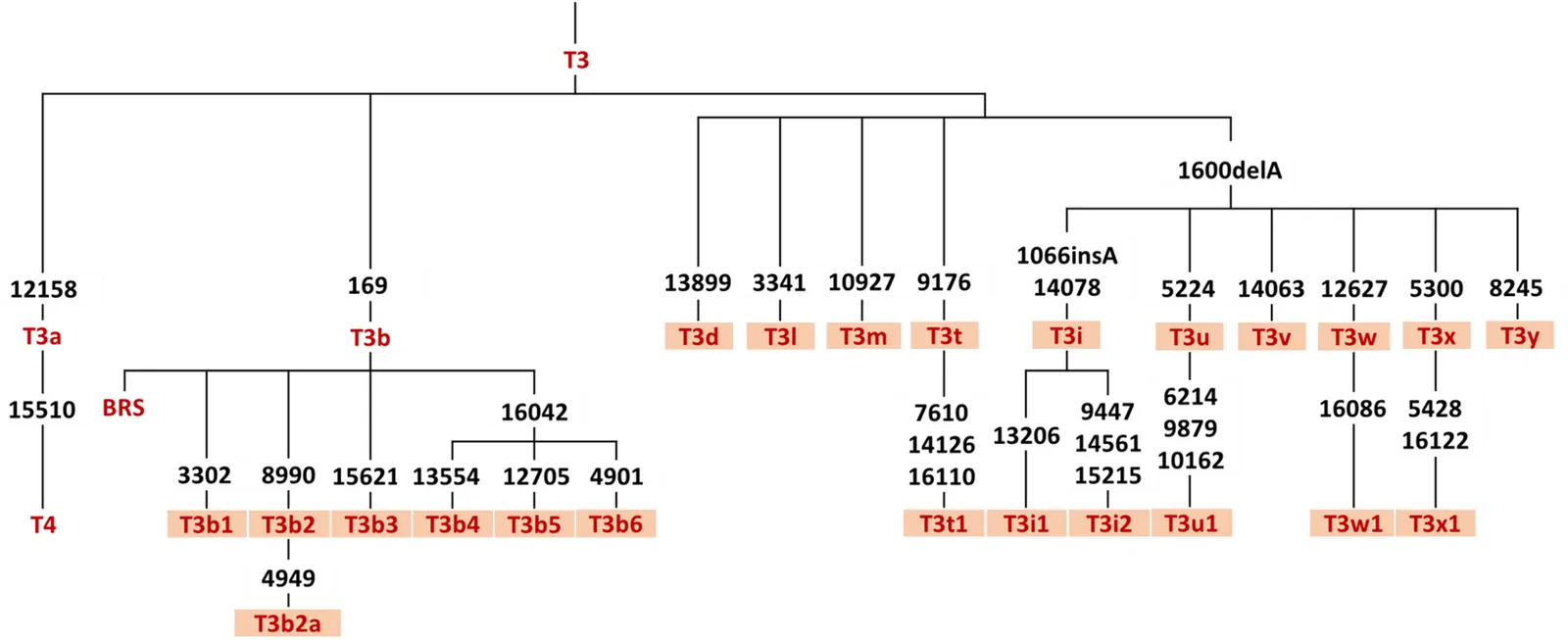
Schematic representation of haplogroup T3 substructure. The 21 novel sub-branches identified here for the first time, as well as two previously described by Dorji and colleagues [6] (T3d and T3l) are shaded in light orange. The mutational motifs (nps) are reported along each branch

Despite the predominance of haplogroup T3 in Europe (Fig. 2), our analyses revealed a remarkably high intra-haplogroup variability among T3 mitogenomes from REN and GAL (Fig. 3a, Additional file 2: Fig. S4). This enabled us to substantially refine the phylogenetic structure of haplogroup T3, through the identification of numerous novel sub-branches, each defined by distinct mutational motifs (Fig. 4, Additional files 1 and 2: Table S3 and Fig. S4). These mutational motifs include both synonymous and nonsynonymous substitutions distributed across coding regions of the mitochondrial genome, with a subset affecting genes involved in oxidative phosphorylation (Additional file 1: Table S3). These new sub-clades were named only if they were formed by at least three distinct haplotypes that shared a specific diagnostic mutational pattern, thus ensuring phylogenetic robustness.

Indeed, within our comprehensive T3 dataset, which includes a total of 634 novel and previously published complete mitogenomes, we identified seven new internal clades within sub-haplogroup T3b (T3b1, T3b2, T3b2a, T3b3, T3b4, T3b5, and T3b6), eight new sister lineages branching alongside T3b (T3m, T3t, T3i, T3u, T3v, T3w, T3x, and T3y) and six lower-level sub-branches (T3t1, T3i1, T3i2, T3u1, T3w1, and T3x1) (Fig. 4, Additional file 1: Table S4). The phylogenetic relationships among the seven T3b internal clades and the T3b sister lineages are reported in Fig. S5 (Additional file 3: Fig. S5). Notably, sub-lineages T3d and T3l, previously identified by Dorji and colleagues [6], were also confirmed in our dataset with a frequency of 0.02 and 0.01, respectively (Additional file 1: Table S4). The resulting structure highlights a previously underappreciated genetic complexity within T3, suggesting multiple regional differentiation events following the initial post-domestication expansion of Near Eastern taurine lineages across Europe and the Mediterranean area.

## Discussion

### MtDNA haplogroup distribution

Surveys of mtDNA variation have provided a powerful framework for understanding the origins and dispersal of taurine domestic cattle (*B. taurus*), tracing their maternal ancestry to a small number of domestication events in the Fertile Crescent approximately 10 ky BP [18, 61, 62]. Recent advances in ancient DNA (aDNA) research have further refined our understanding of cattle domestication dynamics. Moreover, palaeogenomic data indicate that gene flow between domestic taurine cattle and local European aurochs during the 6th − 3rd millennia Before Common Era (BCE) was more frequent and geographically structured than previously thought [37, 63]. Notably, this admixture appears to have primarily affected the nuclear genome, while the mtDNA gene pool largely retained its Near Eastern origin [64, 65]. Therefore, modern European taurine diversity reflects both Near-Eastern founder effects and later regionally structured and recurrent episodes of adaptation and introgression [66].

To place our results in a broader evolutionary framework, it is important to consider the growing body of ancient DNA (aDNA) evidence from Neolithic and Bronze Age cattle across Western Eurasia. Archaeogenetic studies have consistently shown that early domestic herds from Anatolia and the Balkans were already dominated by haplogroup T3, with additional contributions from T2 and the rare Q lineage [31, 67, 68]. These lineages were part of the initial maternal gene pool associated with the domestication process in the Fertile Crescent and its subsequent expansion into Europe [13, 18, 21]. In this context, the predominance of T3 lineages in Rendena and Alpine Grey cattle is fully consistent with the maternal genetic composition of early European domestic herds. At the same time, the detection of T2, T5, and Q1 haplogroups in our dataset suggests the persistence of ancient and geographically structured maternal lineages that were already present in prehistoric cattle populations. Notably, haplogroup Q, although rare in modern breeds, has been identified in Neolithic and Chalcolithic remains from southeastern Europe and Anatolia [67, 68], indicating that its current sporadic distribution likely reflects long-term survival rather than recent introgression. Furthermore, the absence of haplotype sharing between Rendena and other modern breeds, despite phylogenetic proximity, may indicate local differentiation processes acting on ancestral lineages introduced during the early phases of cattle diffusion into the Alpine region. This pattern is consistent with a scenario in which geographically constrained populations, such as those in Alpine valleys, retained unique mtDNA variation while experiencing limited gene flow with surrounding populations.

Haplogroup T3 represents the most widespread T lineage across modern European cattle populations and is extensively regarded as the maternal signature of West-Eurasian taurine herds [7, 18, 69]. Archaeogenetic evidence indicates that T3 lineages accompanied the Neolithic expansion of domestic cattle into Europe, becoming the predominant maternal component of early herds [13, 21, 70]. As these domestic populations expanded westward, they experienced serial founder effects and local differentiation [67]. Indeed, Neolithic cattle remains from Anatolia, the Balkans, southern Germany, and the Alps consistently show a dominance of haplogroup T3 [68]. These patterns provide a relevant framework for interpreting the high frequency and internal diversification of T3 in modern Alpine cattle.

Notwithstanding the predominant presence of T3 among our samples, REN and GAL breeds show a high genetic variability, with many sub-branches defined by private mutations and the detection of rare haplogroups (i.e. T5 and Q1). T1 lineage was absent in the REN and GAL we sampled and in almost all other Alpine cattle breeds (with the exception of one Italian Brown sequence, JN817312) (Additional file 1: Table S2). This indicates that, despite its prevalence in northern Africa, and presence in southern Europe and Near East, Alpine cattle were not subjected to African influence [6, 15, 22, 35].

Ancient DNA studies on Alpine cattle remains (from South Tyrol and the Grisons) dating back to the Bronze Age detected haplotypes from haplogroups T2 and Q, suggesting that these lineages were already well-established in Alpine regions during the second millennium BCE [71]. Our analysis of modern T2 mitogenomes revealed no haplotype sharing between our samples and other breeds, but only a genetic proximity with a sequence from the Dukagjini Busha breed from the Balkans (Additional file 2: Fig. S2). Within haplogroup Q, only one published sequence is close to our sample REN140, differing by the mutation at np 12,480 (Additional file 2: Fig. S1). This sequence derived from the Chianina, an Italian cattle breed from the Tuscany region, of Podolian origin, which seems to have received a dual ancestral contribution from the Balkans and the Middle East [15, 30, 72]. The haplogroup Q1 was detected in two REN and GAL individuals and in few other local breeds from Italy (Alpine Grey, Chianina, Italian Red Pied, and Romagnola), Egypt (Domiaty) and the Iberian Peninsula (Pyrenaica) [10, 18, 21, 24, 27]. Together with the uniqueness of our mtDNA haplotypes, this pattern may indicate an in situ differentiation from the founder domestic populations introduced into these regions. Ancient DNA studies further illuminate the long history of these rare lineages. Haplogroup Q, although scarce today, is associated with Neolithic and Chalcolithic cattle from both Anatolia and southeastern Europe. This suggests that it was part of the initial domesticated maternal pool, but declined rapidly in frequency when Near-Eastern herds spread into Europe [68].

Likewise, haplogroup T5, now concentrated in Alpine and Balkan breeds, has been sporadically identified in Bronze Age cattle from northern Italy and the eastern Alps [71]. Its current fragmented distribution reflects long-term regional persistence rather than recent introgression. The presence of these lineages in REN cattle therefore aligns with a scenario of maternal continuity from early post-domestication populations in the Alpine region. Haplogroup T5 has been previously identified in breeds from the Alpine arc (Original Braunvieh and Valdostana), Northern Italy (Piedmontese) and the Balkans (Skodra Busha and Slavonian Syrmian Podolian) (Additional file 1: Table S2) [6, 18, 27]. Haplogroup T5 diverged approximately 16–18 ky BP [18, 27]. Its current geographic distribution suggests that the REN haplotype (Additional file 2: Fig. S3) derives from mtDNAs already present in the founding populations involved in the domestication event occurred in Anatolia (10–11 ky BP) [24].

The absence of haplogroups P (considered a possible marker of post-glacial auroch expansion from Europe to Asia, and its subsequent incorporation into the early domesticated herds of northeast Asia [34]), E (found in a German Neolithic individual) [73], and R (primarily found in modern Italian cattle breeds) [21] in our samples indicates that the current population of Rendena cattle was not significantly affected by crossbreeding with European aurochs or with cattle deriving from postulated secondary domestication events [19, 21, 24, 33, 35, 67]. This result is consistent with an archaeogenomic study showing that haplogroup P, the dominant lineage in European aurochs, rarely entered the maternal gene pool of domestic cattle [74], despite evidence of significant nuclear introgression in other regions [65].

### Phylogeny and demographic trends

Analyses of Neolithic and Bronze Age samples suggest that domestic populations underwent a sharp female effective population size expansion immediately after domestication, followed by a plateau characterized by regional structuring of herds and increasing human-mediated selection [67]. These temporal patterns provide a comparative framework for interpreting the demographic signatures observed in REN and other Alpine cattle. In fact, the BSP of our REN samples shows changes in effective population size (Ne) over time (Fig. 3b). A marked expansion is observed around 10 ky BP, which is consistent with the post-domestication population growth [27, 75]. However, historical records note that the Rendena represents a modern cattle breed whose current identity and breeding structure have only been developed in recent centuries. Therefore, this signal likely reflects demographic processes occurring in ancestral populations rather than the demographic history of this breed. Following this phase, a plateau of elevated Ne suggests a prolonged period of relatively stable population size, after which a decline is observed around one thousand years ago. More generally, stabilization and decrease phases may reflect complex demographic scenarios rather than a single simple expansion followed by contraction, as revealed by a recent study showing asynchronous expansion patterns among haplogroups (before the domestication for haplogroup T2, 10 − 9.5 kya for haplogroup Q, about 7.5 kya for T3, and 3.0-2.5 kya for T1) [27].

The same broad expansion signal can also be observed in the BSP of Alpine Grey mitogenomes, whereas the later reduction of the effective population size observed in REN is absent in GAL (Fig. 3b). Nevertheless, these differences should not be overinterpreted as breed-specific demographic histories, as both patterns likely reflect variation in the retention of ancestral mtDNA diversity rather than independent demographic trajectories of modern breeds. Comparable patterns have been reported for other local European breeds with restricted maternal diversity [13, 21] and for modern commercial breeds, where human-mediated selection led to reduced mtDNA variability [27]. Moreover, when considering all the Alpine cattle breeds including published mitogenomes (*N* = 241), the BSP shows a trend similar to that of REN, with a Ne decline around 2.5 ky BP (Fig. 3b, Additional file 1: Table S2), indicating shared ancestral dynamics instead of synchronous demographic changes in modern breeds.

Previous studies on bovine mitogenomes have revealed characteristic population dynamics linked to a Neolithic domestication-associated expansion [18, 27, 75] and Middle Holocene post-domestication stabilization [21, 70, 76], indicating a maintenance of Ne while human-managed herds spread and diversified. In general, this pattern might reflect a recent bottleneck consistent with inbreeding, modern isolation and selective pressures causing a reduction of maternal diversity, especially in local, isolated or endangered cattle breeds.

Despite the genetic proximity of the Rendena cattle to breeds from the Alps (Original Braunvieh, Pustertaler Sprinzen and Tux-Zillertaler), Balkans (Boskarin, Croatian Busha, Dukagjini Busha, Skodra Busha and Slavonian Syrmian Podolian), Iberian (Betizuak, Maronesa and Sayaguesa) and Italian (Chianina, Cinisara and Piedmontese) Peninsulas [3, 18, 27] revealed by the comparative analysis with published data, only one mitogenome (MZ901423) belonging to the Tux-Zillertaler, a cattle breed from South-Tyrol, is identical to one Rendena sample (REN186) (Additional file 2: Fig. S4). Moreover, another sequence belonging to the same breed (MZ901424) seems to derive from our REN139, thus gene flow from the Rendena valley towards Austria is also possible. This gene flow was also observed in the internal phylogenetic structure of haplogroup T3 (Additional files 1 and 2: Table S4 and Fig. S4).

### Refinement of haplogroup T3 substructure

Although haplogroup T3 represents the most widespread maternal lineage in European cattle, our analyses indicate that its internal structure is more complex than previously recognized. Rather than a homogeneous haplogroup, T3 can be organized into a limited number of major phylogenetic clusters, within which substantial micro-diversification has occurred. In our dataset, the T3 diversity is primarily structured around the T3b lineage and a series of closely related sister clades, which together account for a large proportion of the observed variation. These major groups likely represent deeply rooted maternal lineages that underwent regional differentiation following the initial spread of domestic cattle across Europe. In addition to these major groups, we also identified six secondary sub-branches (T3t1, T3i1, T3i2, T3u1, T3w1, and T3 × 1) and confirmed the existence of T3d and T3l, first described by Dorji and colleagues [6].

In this phylogenetic framework, the 21 novel sub-clades represent finer-scale branches nested within the major clusters. While these newly defined lineages provide important resolution for reconstructing recent evolutionary relationships, their interpretation is more robust when considered as part of a hierarchical structure organized around a few principal T3 groups, rather than as independent units. From a phylogeographic perspective, the distribution of these major T3 clusters across our dataset and previously published mitogenomes suggests a strong association with Alpine (i.e. Murbodner, Original Braunvieh, and Tux-Zillertaler) and Balkan (i.e. Boskarin, Croatian Busha, Serbian Busha, and Slavonian Syrmian Podolian) cattle breeds (Additional files 1 and 2: Table S4 and Fig. S4), with additional links to breeds from the Italian (i.e. Chianina, Cinisara, Italian Podolian, and Italian Red Pied) and Iberian Peninsulas (i.e. Maronesa and Sayaguesa). This pattern is consistent with regional differentiation processes acting on ancestral lineages introduced during the early phases of cattle diffusion into Europe. The high number of private mutations observed in REN and GAL mitogenomes further supports a scenario of long-term local evolution in geographically constrained populations. These breeds appear to have retained distinct mitochondrial variation while maintaining phylogenetic continuity with broader European lineages. Taken together, our findings demonstrate that haplogroup T3 is a phylogenetically rich and regionally structured assemblage of maternal clades that reflect the complex demographic history of taurine cattle since their domestication.

Beyond their phylogenetic relevance, it is worth considering whether some of the mutations defining the main T3 lineages may also have potential functional implications. Although mitochondrial variation is often interpreted within a neutral evolutionary framework, the mitochondrial genome encodes essential components of the oxidative phosphorylation (OXPHOS) system, and nonsynonymous substitutions may, in some cases, affect protein function or efficiency.

In our dataset, while many variants are synonymous or involve conservative substitutions, others result in physicochemical changes, which may potentially influence protein stability or interactions within the respiratory chain, although their actual functional effects remain to be demonstrated.

These observations should be regarded as preliminary, and further analyses using dedicated predictive tools for mitochondrial variants, such as APOGEE 2 [77], as well as experimental validation, would be required to assess their actual functional impact. Nevertheless, the distribution of nonsynonymous mutations across key OXPHOS genes suggests that mitochondrial diversity in cattle may not be entirely neutral and could contribute, at least in part, to metabolic variation and local adaptation [78].

## Conclusions

The maintenance of local cattle breeds has long been justified as a means of conserving a reservoir of genetic diversity useful for current and future support of traditional and sustainable farming, agrifood products, and resilience in the context of global climate change.

Our findings showed that Rendena cattle in the Province of Trento, Italy, possess a strong maternal connection with a breed from Südtirol/Alto Adige (Alpine Grey) and possibly genetic input from the Balkans. Moreover, the Rendena breed did not seem to have been so much genetically influenced by the importation of bovines from Switzerland after the population decline that it underwent during the rinderpest epidemics that occurred in the 18th Century. Unfortunately, the lack of more complete mitogenome data and ancient DNA samples from the Alps prevents broader comparative analysis. Future studies, particularly on ancient samples, could further elucidate the mtDNA distinctiveness of Rendena cattle.

Our results underscore the importance of the conservation of local breeds as a reservoir of genetic diversity necessary for adaptation to change in farming and climate conditions. In addition, the high haplotype richness and the presence of novel sub-clades identified in the REN and GAL populations highlight the importance of traditional and heritage breeds as genetic reservoirs of lineages that contribute significantly to the understanding of European cattle demographic history and to the conservation of mitochondrial diversity in modern livestock.

The integration of ancient and modern mitochondrial data provides a coherent framework for interpreting Alpine cattle diversity. Current cattle populations retain elements of early Holocene maternal lineages. Rare haplogroups (Q1, T5) and novel T3 sub-lineages further highlight the important role of traditional and geographically constrained breeds, largely erased in intensively selected or globally distributed cattle populations. Continued comparison with expanding ancient-DNA datasets will further elucidate how local demographic processes, post-Neolithic expansions, and historical bottlenecks have shaped present-day mtDNA variation in European taurine cattle.

## Supplementary Information

Below is the link to the electronic supplementary material.

Supplementary Material 1

Supplementary Material 2

Supplementary Material 3

## Data Availability

FastQ data have been deposited at SRA as BioProject PRJNA1381578 and are publicly available as of the date of publication. FASTA data have been deposited in GenBank with accession numbers PX734226-PX734306 and PX734915-PX735001, and are publicly available as of the date of publication. All data are available in the main text or the supplementary materials.
